# Supplementary material for: Joint modeling of choices and reaction times based on Bayesian contextual behavioral control
Source: PLoS Comput Biol. 2024 Jul 5;20(7):e1012228. doi: 10.1371/journal.pcbi.1012228 (PMC11290629; doi:10.1371/journal.pcbi.1012228)
Supplement: S2 Appendix — We explore mathematical relations between the DDM and the BCC. (PDF) [file pcbi.1012228.s002.pdf]

Supplementary file: Relation to DDM

The drift diffusion model (DDM) can be interpreted as an approximation to a sequential probability ratio test [1] which links the DDM to probabilistic processing. Indeed, an equivalence between Bayesian updating and the drift diffusion model can be established for perceptual decision making [2, 3]. Bitzer et al. showed how the quantities in a DDM, e.g. drift rate, bias and boundary, relate to sequential updating of a Bayesian posterior and vice versa [2]. Here, we want to show that a similar equivalence cannot be retrieved for the DDM and the BCC. In short, sequential posterior updates where the posterior becomes the new prior are required to establish the link to the sequential probability ratio test in the DDM. In the BCC however, sampling is used to update or learn Bayesian hyper-parameters, which is a fundamentally different underlying process which leads to a different type of update equation in each step. In what follows we show this in more detail.

In the perceptual decision making model in [2], the authors used a recursive Bayesian update rule to model decision making in a perceptual decision making experiment

$$p\left(a|x_{1:t}\right) = \frac{p\left(x_t|a\right)p\left(a|x_{1:t-1}\right)}{\sum_{a'} p\left(x_t|a'\right)p\left(a'|x_{1:t-1}\right)} \tag{1}$$

where $p\left(x_t|a\right)$ is a Gaussian representing the generative process, and $p\left(a|x_{1:t-1}\right)$ is the previous posterior. The evidence accumulation process starts at $t=0$ with a prior $p\left(a\right) = p_0$ as the previous posterior. The above update is repeated for each observation x_t which updates beliefs about which alternative a explains the observations best, which in turn translates to the currently best action. The authors translated this into an additive update rule for the log odds as

$$q_t = \ln \frac{p\left(a_1|x_{1:t}\right)}{p\left(a_2|x_{1:t}\right)} \tag{2}$$

$$q_t = q_{t-1} + \ln p\left(x_t|a_1\right) - \ln p\left(x_t|a_2\right) \tag{3}$$

and were able to show that this can be mapped to the DDM update rule

$$q_t = q_{t-1} + v\Delta t + \sqrt{\Delta t} s \epsilon_t \tag{4}$$

where Δt is the time in between time steps, v is the classical drift rate in the DDM, s is the diffusion constant, and ϵ_t is the noise term. Using the form equivalence of the two equations, the authors show how exactly these quantities relate to the parameters of the Bayesian model, i.e. means and variances of the Gaussians.

In terms of the MCMC sampling in the BCC, the update rules (Eqs 9-11) have a different form, as they do not equate to a sequential probability ratio test. The sampling process was defined as

$$\pi^* \sim p\left(\pi\right) \tag{5}$$

$$\rho = \min\left\{\frac{p\left(R|\pi^*\right)}{p\left(R|\pi_{n-1}\right)}, 1\right\} \tag{6}$$

$$\pi_n = \begin{cases} \pi^* & \text{with prob. } \rho \\ \pi_{n-1} & \text{with prob. } 1-\rho \end{cases} \tag{7}$$

$$\eta_{\pi,n} = \eta_{\pi,n-1} + \delta_{\pi,\pi_n} \tag{8}$$

$$q\left(\pi|\eta_n\right) = \int d\vartheta_n q\left(\pi|\vartheta_n\right) q\left(\vartheta_n|\eta_n\right) = \frac{\eta_{\pi,n}}{\sum_{\pi'} \eta_{\pi',n}} \tag{9}$$

$$= \frac{\eta_{\pi,n}}{N_\pi + n} \tag{10}$$

In this process, a policy is sampled with a probability according to the prior $p(\pi^*)$ and chosen into the chain with probability according to the likelihood $p\left(R|\pi^*\right)$. Therefore, it can be shown that the probability of a sampled policy π^* being accepted into the chain is proportional to the prior times likelihood, which means that on average (or for larger n) the updates of the Dirichlet parameters can be expressed as:

$$\begin{aligned}\eta_{\pi,n} &= \eta_{\pi,n-1} + p(\pi)\, p\left(R|\pi\right) && (11)\\ &= 1 + n * p(\pi)\, p\left(R|\pi\right) && (12)\end{aligned}$$

which yields the following update equation for the estimated posterior over policies

$$\begin{aligned}q\left(\pi|\eta_n\right) &= \frac{\eta_{\pi,n-1} + p(\pi)\, p\left(R|\pi\right)}{N_\pi + n} && (13)\\ &= \frac{1 + n * p(\pi)\, p\left(R|\pi\right)}{N_\pi + n} && (14)\end{aligned}$$

If this process would correspond to a sequential probability ratio test, the update in the BCC should be form equivalent to the update in the Bayesian model in [2]. However, the update in [2]is additive in log space (as it is multiplicative in probability space due to its sequential nature), whereas the update in the BCC is additive in probability space. Hence, the two represent different types of processes and the BCC can not be mapped to the DDM in the same way.

Indeed, also a visual comparison of our Figure 4 and Figure 5a in [2] shows a key difference: In the BCC, the probability of the posterior approaches its true value, but oscillates around it, with decreasing amplitude as the process gets closer to the stopping criterion. In the model of [2], the posterior approaches the boundary from below but never crosses it.

However, we expect that that certain properties are shared, like the influence of the certainty in the prior over policies or the strength of the evidence in the likelihood.

References

[1] Bogacz R, Brown E, Moehlis J, Holmes P, Cohen JD. The physics of optimal decision making: a formal analysis of models of performance in two-alternative forced-choice tasks. Psychological review. 2006;113(4):700.

[2] Bitzer S, Park H, Blankenburg F, Kiebel SJ. Perceptual decision making: drift-diffusion model is equivalent to a Bayesian model. Frontiers in human neuroscience. 2014;8:102.

[3] Fard PR, Park H, Warkentin A, Kiebel SJ, Bitzer S. A Bayesian reformulation of the extended drift-diffusion model in perceptual decision making. Frontiers in computational neuroscience. 2017;11:29.
